# Crystal structure of calcium perchlorate anhydrate, Ca(ClO_4_)_2_, from laboratory powder X-ray diffraction data

**DOI:** 10.1107/S2056989018003936

**Published:** 2018-03-09

**Authors:** Dongmin Lee, Hyeri Bu, Dohwan Kim, Jooeun Hyoung, Seung-Tae Hong

**Affiliations:** a Daegu Gyeongbuk Institute of Science and Technology (DGIST), Daegu 42988, Republic of Korea

**Keywords:** crystal structure, powder X-ray diffraction, calcium perchlorate anhydrate, Ca(ClO_4_)_2_, isotypism

## Abstract

The crystal structure of calcium perchlorate anhydrate was determined from laboratory X-ray powder diffraction data. The title compound was obtained by heating hydrated calcium perchlorate [Ca(ClO_4_)_2_·*x*H_2_O] at 623 K in air for 12 h. It crystallizes in the ortho­rhom­bic space group *Pbca* and is isotypic with Ca(AlD_4_)_2_. The asymmetric unit contains one Ca, two Cl and eight O sites, all on general sites (Wyckoff position 8*c*). The crystal structure consists of isolated ClO_4_^−^ tetra­hedra and Ca^2+^ cations. The Ca^2+^ cation is coordinated by eight O atoms of eight symmetry-related ClO_4_^−^ tetra­hedra within a distorted square-anti­prismatic environment.

## Abstract 2

The crystal structure of anhydrous Ca(ClO_4_)_2_ crystallizes isotypically with Ca(AlD_4_)_2_.

## Chemical context   

Recently, the alkaline earth metals, in particular magnesium and calcium, have received attention because of their incorporation in multivalent-ion batteries that can replace Li-ion batteries (Wang *et al.*, 2013 ▸; Datta *et al.*, 2014 ▸; Amatucci *et al.*, 2001 ▸). Calcium has several merits, such as low cost and abundance in nature (Padigi *et al.*, 2015 ▸; Rogosic *et al.*, 2014 ▸). In addition, the standard reduction potential of the calcium electrode is −2.87 V, which is only about 0.18 V higher than that of lithium (Muldoon *et al.*, 2014 ▸). Thus, calcium perchlorate is mainly used as a salt next to organic electrolytes in Ca-ion batteries (Hayashi *et al.*, 2003 ▸). Nevertheless, the crystal structure of anhydrous calcium perchlorate was unknown until now (Pearse & Pflaum, 1959 ▸) because of the lack of single crystals. Calcium perchlorate is strongly hygroscopic, and growing single crystals of a size sufficient for X-ray structure analysis has not been successful up to date. On the other hand, the crystal structures of the perchlorates of magnesium, barium and other alkaline earth metals have been determined for both hydrated and anhydrous phases (Gallucci & Gerkin, 1988 ▸; Lee *et al.*, 2015 ▸; Lim *et al.*, 2011 ▸; Robertson & Bish, 2010 ▸). However, for calcium perchlorate only the hydrated forms were structurally determined (Hennings *et al.*, 2014 ▸).

**Figure 1:**
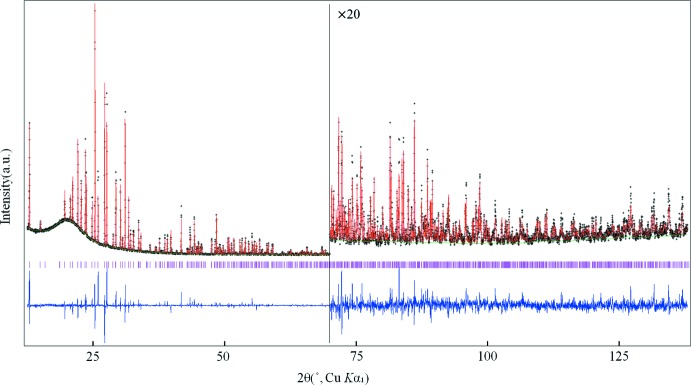
PXRD Rietveld refinement profiles for anhydrous Ca(ClO_4_)_2_ measured at ambient temperature. Crosses mark experimental data (black), the solid red line represents the calculated profile (red) and the solid green line is the background. The bottom trace represents the difference curve (blue) and the ticks denote the positions of expected Bragg reflections (magenta).

We present here the crystal structure of calcium perchlorate anhydrate, using laboratory powder X-ray diffraction (PXRD) data (Fig. 1 ▸).

## Structural commentary   

**Figure 2:**
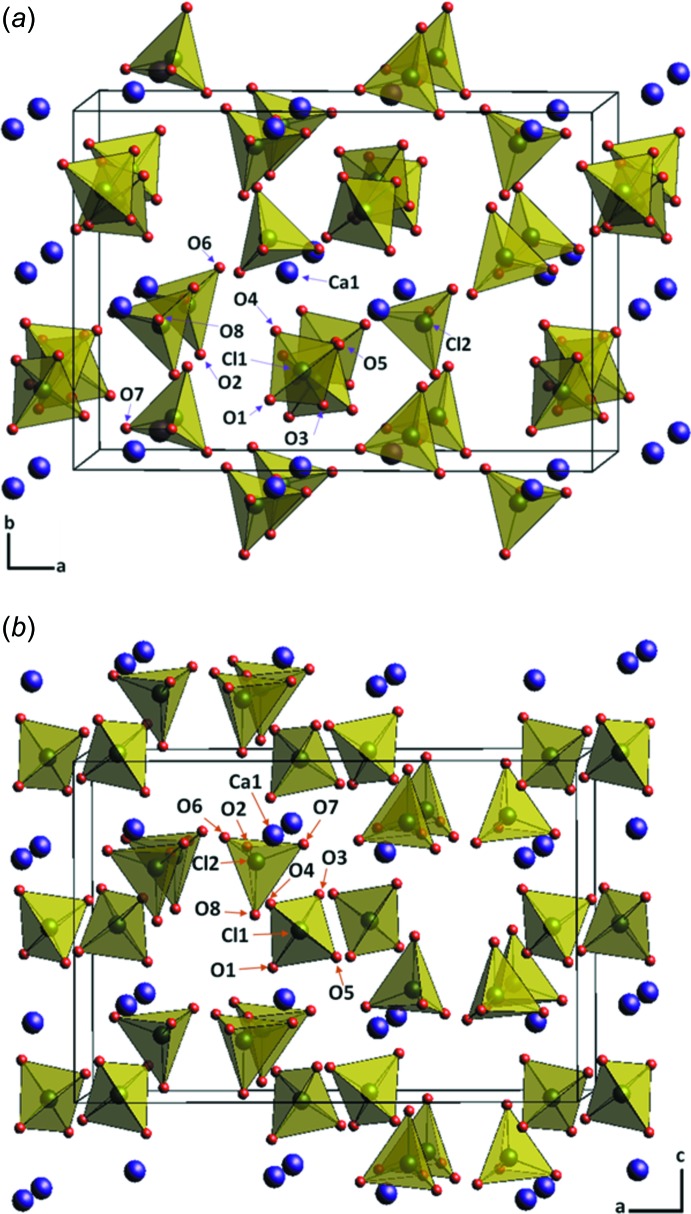
The crystal structure of Ca(ClO_4_)_2_ with ClO_4_^−^ tetra­hedra (yellow) and Ca^2+^ cations (purple), showing (*a*) a view approximately along [001] and (*b*) approximately along [010].

**Figure 3:**
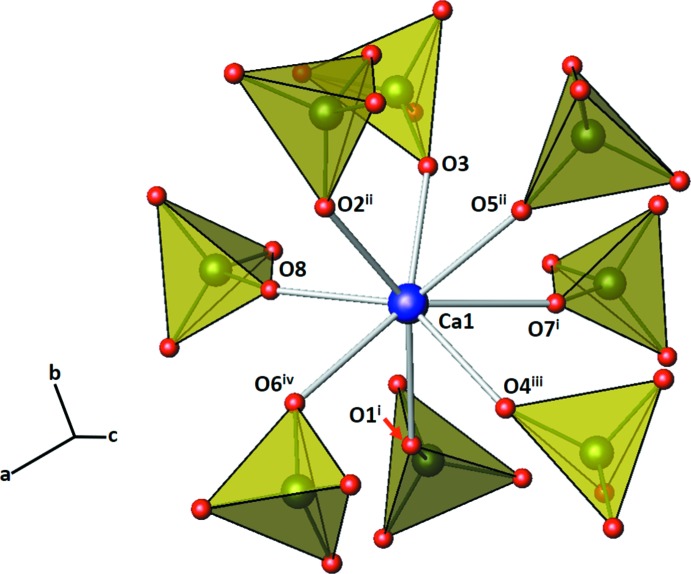
The local environment of the Ca^2+^ cation (purple sphere) surrounded by ClO_4_^−^ tetra­hedra (yellow). [Symmetry codes: (i) −*x* + 1, −*y* + 1, −*z* + 1; (ii) *x*, −*y* + 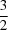, *z* + 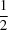; (iii) −*x* + 1, *y* − 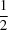, −*z* + 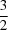; (iv) −*x* + 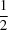, −*y* + 1, *z* + 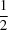.]

The crystal structure of anhydrous calcium perchlorate, Ca(ClO_4_)_2_, is isotypic with that of Ca(AlD_4_)_2_ (Sato *et al.*, 2009 ▸), but is different from barium or magnesium perchlorates (Lee *et al.*, 2015 ▸; Lim *et al.*, 2011 ▸). Different viewing directions of the crystal structure of Ca(ClO_4_)_2_ are presented in Fig. 2 ▸, using ClO_4_^−^ tetra­hedra and Ca^2+^ cations. The unit cell contains one Ca (on general positions 8*c*), two Cl (8*c*), and eight O (8*c*) sites. The ClO_4_^−^ tetra­hedra are slightly distorted [mean Cl—O distance 1.43 (2) Å, angular range 103.5 (4)–114.6 (4)°] and isolated from each other. The local environment around the Ca^2+^ cation is presented in Fig. 3 ▸. It is coordinated by eight isolated ClO_4_^−^ tetra­hedra with an apex oxygen atom of each tetra­hedron bonded to the Ca^2+^ cation. The resulting coordination sphere can be considered as a distorted square anti­prism. The average Ca—O distance is 2.476 Å (Table 1 ▸), which is inter­mediate between those of comparable Mg—O (2.098 Å) and Ba—O (2.989 Å) polyhedra (Lee *et al.*, 2015 ▸; Lim *et al.*, 2011 ▸), and consistent with the sum of the ionic radii of the alkaline earth metals and oxygen (Shannon, 1976 ▸). The coordination number of the Mg^2+^, Ca^2+^, and Ba^2+^ cations in the anhydrous perchlorates increases from 6, 8, and to 12, respectively.

## Synthesis and crystallization   

In order to prepare calcium perchlorate anhydrate, Ca(ClO_4_)_2_·*x*H_2_O (reagent grade, Alfa Aesar) was placed in 75 ml glass vials. The vials were placed into a box furnace, heated at 623 K for 12 h with a heating rate of 3 K min^−1^, cooled down to 423 K, and transferred to a glove box under an Ar atmosphere. The exposed time in a normal atmosphere during the transfer was about 10 s. The sample was ground using an agate mortar, and placed in a dome-type PXRD sample holder that was sealed tightly to prevent atmospheric exposure during the data collection.

## Refinement details   

Crystal data, data collection and structure refinement details are summarized in Table 2 ▸. The powder XRD data of anhydrous calcium perchlorate were collected using a Bragg–Brentano diffractometer (PANalytical Empyrean) with Cu *K*α_1_ radiation (λ = 1.5406 Å) at 40 kV and 30 mA, using a graphite monochromator and a Pixcel3D 2×2 detector. X-ray intensities were measured for 12 h at 0.013° inter­vals in the angular range of 5° ≤ 2*θ* ≤ 140°. X-ray diffraction data were indexed by the *TREOR90* algorithm (Werner, 1990 ▸) in the *CRYSFIRE* program suite (Shirley, 2002 ▸), with 22 indexed reflections starting from the smallest angle. An ortho­rhom­bic unit cell was revealed suggesting *Pbca* as the most probable space group. Based on these results, the refinement process was performed using the *GSAS* program (Larson & Von Dreele, 2000 ▸) and the *CRYSTALS* program (Betteridge *et al.*, 2003 ▸). The process was started with the assumption that there is one dummy atom at an arbitrary position. Then direct methods were applied to calculate the initial solution of the crystal structure using *SHELXS97* (Sheldrick, 2008 ▸), which yielded a Ca site as a starting postition. The initial model was then replaced with the partial model, and this data was used for a LeBail fit in *GSAS*. Then, improved structure factors were calculated, which were used for the refinement in *CRYSTALS*. These processes were repeated until a complete and sufficient structural model converged. Based on these results, the *MCE* programme (Rohlíček & Hušák, 2007 ▸) was used to draw the calculated Fourier-density map in three dimensions. For the final Rietveld refinement with *GSAS*, an overall displacement parameter was used, and Cl—O bond lengths were restrained with a tolerance value of 25% from the distances determined from *CRYSTALS*, where the distances matched well with Shannon’s radii sum. Pseudovoigt profile coefficients as parameterized in Thompson *et al.* (1987 ▸), asymmetry correction of Finger *et al.* (1994 ▸) and microstrain broadening of Stephens (1999 ▸).

## Supplementary Material

Crystal structure: contains datablock(s) I. DOI: 10.1107/S2056989018003936/wm5437sup1.cif

Supporting information file. DOI: 10.1107/S2056989018003936/wm5437Isup2.cml

CCDC reference: 1827999

Additional supporting information:  crystallographic information; 3D view; checkCIF report

## Figures and Tables

**Table 1:** Selected bond lengths (Å)

Ca1—O1^i^	2.451 (6)	Cl1—O2	1.411 (6)
Ca1—O2^ii^	2.412 (6)	Cl1—O6	1.414 (6)
Ca1—O3	2.448 (6)	Cl1—O7	1.421 (6)
Ca1—O4^iii^	2.370 (6)	Cl1—O8	1.423 (6)
Ca1—O5^ii^	2.429 (6)	Cl2—O1	1.456 (6)
Ca1—O6^iv^	2.512 (6)	Cl2—O3	1.408 (6)
Ca1—O7^i^	2.519 (6)	Cl2—O4	1.453 (6)
Ca1—O8	2.413 (6)	Cl2—O5	1.442 (6)

Symmetry codes: (i) 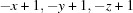; (ii) 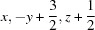; (iii) 
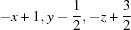
; (iv) 
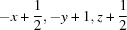
.

**Table 2:** Experimental details

Crystal data	
Chemical formula	Ca(ClO_4_)_2_
*M*_r_	238.98
Crystal system, space group	Orthorhombic, *P* *b* *c* *a*
Temperature (K)	295
*a*, *b*, *c* (Å)	13.75102 (8), 9.50887 (5), 9.06168 (5)
*V* (Å^3^)	1184.88 (1)
*Z*	8
Radiation type	Cu *K*α_1_, λ = 1.5405 Å
Specimen shape, size (mm)	Flat sheet, 24.9 × 24.9

Data collection	
Diffractometer	PANalytical Empyrean
Specimen mounting	Packed powder
Data collection mode	Reflection
Scan method	Step
2θ values (°)	2θ_min_ = 5.001 2θ_max_ = 139.993 2θ_step_ = 0.013

Refinement	
*R* factors and goodness of fit	*R*_p_ = 0.068, *R*_wp_ = 0.104, *R*_exp_ = 0.055, *R*(*F* ^2^) = 0.151, χ^2^ = 3.610
No. of parameters	44

Computer programs: *X’Pert Data Collector* and *X’Pert HighScore Plus* (PANalytical, 2011 ▸), *GSAS* (Larson & Von Dreele, 2000 ▸), *SHELXS97* (Sheldrick, 2008 ▸), *CRYSTALS* (Betteridge *et al.*, 2003 ▸) and *ATOMS* (Dowty, 2000 ▸).

## supplementary crystallographic information

### Crystal data

**Table:**

Ca(ClO_4_)_2_	*Z* = 8
*M**_r_* = 238.98	*F*(000) = 944.0
Orthorhombic, *P**b**c**a*	*D*_x_ = 2.680 Mg m^−^^3^
Hall symbol: -P_2ac_2ab	Cu *K*α_1_ radiation, λ = 1.5405 Å
*a* = 13.75102 (8) Å	*T* = 295 K
*b* = 9.50887 (5) Å	white
*c* = 9.06168 (5) Å	flat_sheet, 24.9 × 24.9 mm
*V* = 1184.88 (1) Å^3^	Specimen preparation: Prepared at 295 K

### Data collection

**Table:**

PANalytical Empyrean diffractometer	Data collection mode: reflection
Radiation source: sealed X-ray tube, PANalytical Cu Ceramic X-ray tube	Scan method: step
Specimen mounting: packed powder	2θ_min_ = 5.001°, 2θ_max_ = 139.993°, 2θ_step_ = 0.013°

### Refinement

**Table:**

Least-squares matrix: full	Excluded region(s): 5 to 12.5 degrees are excluded due to background scattering at low angles, in addition there are no peaks in this region.
*R*_p_ = 0.068	Profile function: CW Profile function number 4 with 18 terms Pseudovoigt profile coefficients as parameterized in P. Thompson, D.E. Cox & J.B. Hastings (1987). J. Appl. Cryst.,20,79-83. Asymmetry correction of L.W. Finger, D.E. Cox & A. P. Jephcoat (1994). J. Appl. Cryst.,27,892-900. Microstrain broadening by P.W. Stephens, (1999). J. Appl. Cryst.,32,281-289. #1(GU) = 9.638 #2(GV) = -11.095 #3(GW) = 2.275 #4(GP) = 4.393 #5(LX) = 0.935 #6(ptec) = 0.00 #7(trns) = 0.00 #8(shft) = -4.2154 #9(sfec) = 0.00 #10(S/L) = 0.0005 #11(H/L) = 0.0005 #12(eta) = 0.7500 #13(S400 ) = 0.0E+00 #14(S040 ) = 0.0E+00 #15(S004 ) = 0.0E+00 #16(S220 ) = 0.0E+00 #17(S202 ) = 0.0E+00 #18(S022 ) = 0.0E+00 Peak tails are ignored where the intensity is below 0.0100 times the peak Aniso. broadening axis 0.0 0.0 1.0
*R*_wp_ = 0.104	44 parameters
*R*_exp_ = 0.055	0 restraints
*R*(*F*^2^) = 0.15096	(Δ/σ)_max_ = 0.04
10385 data points	Background function: GSAS Background function number 1 with 36 terms. Shifted Chebyshev function of 1st kind 1: 396.859 2: -606.961 3: 459.581 4: -240.760 5: 60.9683 6: 66.1787 7: -127.055 8: 123.403 9: -80.0454 10: 22.9955 11: 31.6319 12: -68.9521 13: 82.3967 14: -74.9306 15: 52.4628 16: -22.9755 17: -7.07207 18: 29.6007 19: -41.2483 20: 39.7866 21: -28.2300 22: 12.3296 23: 2.74056 24: -14.4441 25: 20.2978 26: -20.5325 27: 15.0728 28: -6.57858 29: -1.96745 30: 7.61710 31: -10.5263 32: 10.4139 33: -6.95249 34: 2.74624 35: 0.930279 36: -1.93129

### Fractional atomic coordinates and isotropic or equivalent isotropic displacement parameters (Å^2^)

**Table:**

	*x*	*y*	*z*	*U*_iso_*/*U*_eq_	
Ca1	0.39788 (14)	0.5357 (2)	0.7164 (2)	0.0110 (2)*	
Cl1	0.34080 (17)	0.6066 (3)	0.3157 (3)	0.0110 (2)*	
Cl2	0.55928 (18)	0.7776 (3)	0.4961 (3)	0.0110 (2)*	
O1	0.6154 (4)	0.7025 (6)	0.3850 (6)	0.0110 (2)*	
O2	0.3176 (4)	0.7464 (6)	0.2773 (6)	0.0110 (2)*	
O3	0.5240 (4)	0.6775 (7)	0.5973 (7)	0.0110 (2)*	
O4	0.6137 (4)	0.8834 (6)	0.5773 (6)	0.0110 (2)*	
O5	0.4842 (4)	0.8546 (7)	0.4199 (6)	0.0110 (2)*	
O6	0.2815 (4)	0.5078 (6)	0.2414 (6)	0.0110 (2)*	
O7	0.4359 (4)	0.5744 (6)	0.2647 (7)	0.0110 (2)*	
O8	0.3387 (4)	0.5833 (6)	0.4708 (7)	0.0110 (2)*	

### Geometric parameters (Å, º)

**Table:**

Ca1—Cl1	3.776 (3)	Cl2—Ca1	3.769 (3)
Ca1—Cl1^i^	3.605 (3)	Cl2—Ca1^v^	3.808 (3)
Ca1—Cl1^ii^	3.662 (3)	Cl2—Ca1^iii^	3.596 (3)
Ca1—Cl1^iii^	3.851 (3)	Cl2—Ca1^vii^	3.627 (3)
Ca1—Cl2	3.769 (3)	Cl2—O1	1.456 (6)
Ca1—Cl2^i^	3.808 (3)	Cl2—O3	1.408 (6)
Ca1—Cl2^iii^	3.596 (3)	Cl2—O4	1.453 (6)
Ca1—Cl2^iv^	3.627 (3)	Cl2—O5	1.442 (6)
Ca1—O1^iii^	2.451 (6)	O1—Ca1^iii^	2.451 (6)
Ca1—O2^i^	2.412 (6)	O1—Cl2	1.456 (6)
Ca1—O3	2.448 (6)	O2—Ca1^v^	2.412 (6)
Ca1—O4^iv^	2.370 (6)	O2—Cl1	1.411 (6)
Ca1—O5^i^	2.429 (6)	O3—Ca1	2.448 (6)
Ca1—O6^ii^	2.512 (6)	O3—Cl2	1.408 (6)
Ca1—O7^iii^	2.519 (6)	O4—Ca1^vii^	2.370 (6)
Ca1—O8	2.413 (6)	O4—Cl2	1.453 (6)
Cl1—Ca1	3.776 (3)	O5—Ca1^v^	2.429 (6)
Cl1—Ca1^v^	3.605 (3)	O5—Cl2	1.442 (6)
Cl1—Ca1^vi^	3.662 (3)	O6—Ca1^vi^	2.512 (6)
Cl1—Ca1^iii^	3.851 (3)	O6—Cl1	1.414 (6)
Cl1—O2	1.411 (6)	O7—Ca1^iii^	2.519 (6)
Cl1—O6	1.414 (6)	O7—Cl1	1.421 (6)
Cl1—O7	1.421 (6)	O8—Ca1	2.413 (6)
Cl1—O8	1.423 (6)	O8—Cl1	1.423 (6)
			
O1^iii^—Ca1—O2^i^	147.7 (2)	O6^ii^—Ca1—O8	77.5 (2)
O1^iii^—Ca1—O3	113.4 (2)	O7^iii^—Ca1—O8	116.53 (2)
O1^iii^—Ca1—O5^i^	135.9 (2)	O2—Cl1—O6	112.2 (4)
O1^iii^—Ca1—O6^ii^	79.0 (2)	O2—Cl1—O7	109.4 (4)
O1^iii^—Ca1—O7^iii^	73.10 (19)	O2—Cl1—O8	112.7 (4)
O1^iii^—Ca1—O8	78.62 (18)	O6—Cl1—O7	103.5 (4)
O2^i^—Ca1—O3	87.3 (2)	O6—Cl1—O8	110.8 (4)
O2^i^—Ca1—O5^i^	71.4 (2)	O7—Cl1—O8	107.8 (4)
O2^i^—Ca1—O6^ii^	70.80 (19)	O1—Cl2—O3	107.6 (4)
O2^i^—Ca1—O7^iii^	139.2 (2)	O1—Cl2—O4	114.6 (4)
O2^i^—Ca1—O8	84.0 (2)	O1—Cl2—O5	107.3 (4)
O3—Ca1—O5^i^	75.6 (2)	O3—Cl2—O4	108.4 (4)
O3—Ca1—O6^ii^	145.7 (2)	O3—Cl2—O5	114.1 (4)
O3—Ca1—O7^iii^	67.4 (2)	Ca1^iii^—O1—Cl2	132.3 (4)
O3—Ca1—O8	74.3 (2)	Ca1^v^—O2—Cl1	139.7 (4)
O5^i^—Ca1—O6^ii^	118.8 (2)	Ca1—O3—Cl2	154.6 (4)
O5^i^—Ca1—O7^iii^	71.6 (2)	Ca1^v^—O5—Cl2	158.7 (4)
O5^i^—Ca1—O8	141.7 (2)	Ca1^vi^—O6—Cl1	135.9 (4)
O6^ii^—Ca1—O7^iii^	144.7 (2)	Ca1^iii^—O7—Cl1	154.5 (4)

Symmetry codes: (i) *x*, −*y*+3/2, *z*+1/2; (ii) −*x*+1/2, −*y*+1, *z*+1/2; (iii) −*x*+1, −*y*+1, −*z*+1; (iv) −*x*+1, *y*−1/2, −*z*+3/2; (v) *x*, −*y*+3/2, *z*−1/2; (vi) −*x*+1/2, −*y*+1, *z*−1/2; (vii) −*x*+1, *y*+1/2, −*z*+3/2.
